# Response to a Specific and Digitally Supported Training at Home for Students With Mathematical Difficulties

**DOI:** 10.3389/fpsyg.2020.02039

**Published:** 2020-08-31

**Authors:** Anna Maria Re, Silvia Benavides-Varela, Martina Pedron, Maria Antonietta De Gennaro, Daniela Lucangeli

**Affiliations:** ^1^Department of Psychology, University of Turin, Turin, Italy; ^2^Department of Developmental Psychology and Socialisation and Department of Neuroscience, University of Padua, Padua, Italy; ^3^Fondazione Opera Edimar, Padua, Italy; ^4^Department of Developmental Psychology and Socialisation, University of Padua, Padua, Italy

**Keywords:** mathematical difficulty, digital training, students, home training, app

## Abstract

The present study evaluated the effectiveness of a shortened, specialized, and digitally supported training program for enhancing numerical skills in primary and secondary school children with mathematical difficulty (MD). The participants (*n* = 57) were randomly assigned to two groups: for the experimental group, the tasks were differentiated and adapted to each student’s learning profile. Moreover, children of this group used a Web App (i.e., “I bambini contano” or “Children count” in English) for improving arithmetic fact retrieval at home; for the control group, the difficulty of the activities was graded according to the school curriculum, and this group did not use the Web App. Pre‐ to post-training measurements showed that children of the experimental group had an improvement significantly higher than the control group, in particular in arithmetic facts and written calculation. Moreover, a follow-up evaluation indicated that the efficacy of the experimental training program lasted up to 2 months after the intervention. The results indicate that a specialized face-to-face intervention along with a digitally supported training at home can benefit children with mathematical learning difficulties.

## Introduction

Approximately 3–8% of school-age children have a diagnosis of mathematical difficulty (MD; Shalev et al., 2000; Desoete et al., 2004; Nelson and Powell, 2017). These students obtain poor results in mathematics, are at risk for math failure, and have difficulty performing several tasks, such as processing number sets, counting, and fact retrieval (Geary et al., 2012). MD is associated with problems at school and even in activities of daily living later in adulthood (e.g., Semenza et al., 2014; Arcara et al., 2017; Benavides-Varela et al., 2020b). Mathematical competence accounts for variance in employment, income, and occupational productivity (Rivera-Batiz, 1992).

Students with MD consistently performed lower than typically developing children across skills (i.e., counting, retrieving facts, fraction comparison and estimation, and applied problem-solving). From a recent review of the literature (Nelson and Powell, 2017), it emerges that targeted interventions and early recognition of math difficulty are fundamental in order to reduce the risk of poor secondary and adulthood outcomes, thus highlighting the importance of addressing children’s math deficits as early as possible. As noted by the review, with appropriate and specific mathematics interventions, even at later stages, students with MD are likely to improve their math performance (Geary et al., 2010; Re et al., 2014).

Although early prevention strategies can substantially reduce the extent of math difficulties (e.g., Fuchs et al., 2005; Bryant et al., 2011), there is no program that appears to be universally effective.

A meta-analysis of mathematics intervention research on students with MD identified four intervention approaches: providing teachers or students with data and feedback on children’s mathematics performance, peer-assisted learning, parental support, and explicit or contextualized teacher-facilitated instruction (Baker et al., 2002). The authors found that the peer-assisted learning approach had the largest effects, followed by explicit instruction and giving student’s data and feedback. By contrast, contextualized teacher-facilitated instruction yielded an overall effect size (ES) that was near to zero.

In the more recent meta-analysis (Dennis et al., 2016), involving 25 studies published between 2000 and 2014, the authors included several variables that might have influenced the outcomes of the training programs: participant characteristics (e.g., age, grade level, etc.), intervention parameters (instructional grouping, intervention agent, and duration of the intervention), domain of intervention, intervention approaches according to the categories defined by Baker et al. (2002), quality of research methodology as categorized by Gersten et al. (2005), and intervention components according to Swanson et al. (1999). The authors found that mathematics interventions had an overall positive impact on math performance of students with MD. Interventions were much more effective when provided by the researchers or a researcher-trained graduate assistant than when provided by teachers or paraprofessionals. There were no significant differences between instructions provided to small groups or on a one-to-one basis; lastly, controlling task difficulty significantly and positively predicted ES estimates. Moreover, the authors underlined the lack of studies on interventions at secondary level.

Another point highlighted by the meta-analysis was the effectiveness of controlling task difficulty, by sequencing tasks from easy to difficult. Accordingly, in a previous study (Re et al., 2014), we compared two types of training – specific empowerment versus a more general scholastic training – for children with different levels of mathematical difficulties (children with dyscalculia or with math difficulties). The specific empowerment training was tailored to the children’s mathematical and cognitive profiles. Moreover, task difficulty was graded in such a way that all the activities were carefully sequenced from easy to difficult for each single child. The results showed that students in the specific empowerment training condition performed better than those following the general scholastic support, both immediately after training and at a later follow-up assessment. These results, in line with the findings of a recent meta-analysis (Jitendra et al., 2018), underlined the importance of choosing appropriate intervention design, materials, strategies, and adequate instructional time (the authors suggest >10 h).

Another important issue in the field of mathematics training is the use of emerging technologies, such as computer and mobile applications (Chodura et al., 2015). A recent meta-analysis including randomized controlled studies indicated a positive effect of digital-based interventions for children with mathematical learning difficulties. The positive effects were observed when interventions employed ludic video games that require the student to indirectly apply numerical concepts to proceed in the game and eventually win, and also with digital-based tutorials/drilling that explicitly instruct mathematical concepts and exercises on a given topic. Moreover, similar gains were observed for children in preschool and primary school, even if the authors suggest that there is still heterogeneity across studies (Benavides-Varela et al., 2020a).

One specific example showing the contribution of technology in teaching mathematics *via* digital tutoring and drilling is the study by Burns et al. (2012). The authors proposed a computer-based intervention to 216 third‐ and fourth-grade students who were at risk for math difficulties and compare it to those of 226 students in a control group. The experimental intervention implemented a computer software to exercise math facts on an average of three times per week for 8–15 weeks. The study reported significantly larger gains among the students who took part in the intervention program compared to those in the control group. Another study (Fuchs et al., 2006) employed computer-assisted instruction (CAI) to train number combination skills among children at risk for math and reading disabilities. Students were assigned randomly to math or spelling CAI, which they received in 50 sessions over 18 weeks. The results indicated that math CAI was effective in promoting addition but not subtraction number combination skills.

There are encouraging results also for kindergarten children, which may contribute at reducing the risk of developing mathematics problems in the future (e.g., Salminen et al., 2015; Sella et al., 2016; Aunio and Mononen, 2018). For example Aragón‐Mendizábal et al. (2017) improved number sense in a sample of 156 kindergarten children using a software that trains mathematical reasoning. The software includes several activities, such as classification, comparisons, distribution, discrimination, seriation, number line tasks, and introduction to addition and subtraction problem solving. Children in the experimental group followed 35 intervention sessions with the software, 30 min each three times per week. Results showed that they outperformed children of a control group, demonstrating that the software was effective even at kindergarten level.

Less is known about the use of mobile applications in mathematics training, even if there is growing evidence that such technological tools also have a positive impact in teaching mathematics (e.g., Baker et al., 2018). Some examples are: MathBoard, a math app appropriate both for kindergarten children using basic addition and subtraction problems, and for primary school students, using multiplication and division tasks, squares, cubes, and square root problems; the “Long Division” app, where students can study and practice the long division method, solving problems step by step (Drigas et al., 2016); and a very recent App designed by Mera et al. (2019) for children from 4 to 7 years old. The App intends to train comparison of magnitudes, subitazing, numerical facts, and estimation on the number line.

In one study, Kiger et al. (2012) employed a mobile learning intervention (MLI) for 87 third-grade students. Two classes used an App named Everyday Math and daily practice using flashcards, etc., to learn multiplication. Two other classes used Everyday Math and web applications for the iPod touch for daily practice. The MLI group outperformed students in the comparison group on a post-intervention multiplication test; indeed, the students who took part in the MLI intervention did many more correct multiplications than their peers in the control group.

O’Malley et al. (2013) presented a 4-week study conducted in a class of 10 children (12–15 years old) with cognitive disabilities at a special education school. The study was designed to examine the effect of using iPads to increase math fluency. According to the results of the study, the iPad was a useful learning tool that helped to reinforce the teachers’ learning intentions and foster the students’ interest in the educational process.

The above-mentioned studies highlight the importance of identifying the most effective strategies and teaching practices to support children with dyscalculia or MD, but there is still little general consensus, or any specific and clear guidelines, on how to proceed. The use of technology creates an accessible and interactive environment that enables personalized learning and helps students with mathematical difficulties to improve their performance. However, there is definitely the need for further research to understand the benefits of using technologies as a part of the educational process (Drigas et al., 2016).

The purpose of the present study was therefore to make a contribution in this field, by investigating the role of a Web App (“I bambini contano”/in English “*Children count*”) in a specific training program for students with MD. Starting from the results of previous studies, according to which a training program has to be tailored on the basis of the student’s mathematics learning profile in order to be effective (Re et al., 2014), we have developed a Web App for arithmetic facts that allows children to practice on their own at home and reduce the number of sessions they have to attend followed by an specialist. Indeed, the principal limitation of the previous study was the length of the intervention period (48 sessions). Our hypothesis is that, maintaining the structure of the training program with activities tailored on the basis of the child’s mathematical learning profile and also using the Web App “I bambini contano” for practicing arithmetic facts at home, we could obtain positive improvements in several mathematical areas and especially in arithmetic facts, and at the same time reducing the number of face-to-face sessions (therefore, we proposed 30 sessions instead of 48, as in our previous study, Re et al., 2014).

In particular, our research questions are:

1. How effective is a specific training program with the Web App “I bambini contano” used at home compared to a general training program for children with MD?
2. What qualitative changes are produced by the two types of training?
3. Did the Web App make a specific contribution to the training program, for example, by improving the children’s ability to retrieve arithmetic facts?
4. To what extent were the effects of the training program maintained 2 months after it ended?

## Materials and Methods

### Participants

The sample consisted of 57 students from primary and secondary schools (see a summary of the demographic data in Table 1 and the individual values in Supplementary Table A). All the participants were attended and were recruited at one of eight centers specialized in the assessment, prevention, and rehabilitation of learning disabilities in Northern Italy, i.e., “Polo Apprendimento,” where they had received the diagnosis of MD. The assessment was carried out by either a child psychiatrist or a clinical psychologist in accordance with the guidelines provided by the Consensus Conference (2010) on Learning Disabilities and the DSM-5 criteria (American Psychiatric Association, 2013). The standardized assessment certified the presence of mathematical skills below those expected for the individual’s chronological age, which cause significant interference with academic or occupational performance or with activities of daily living. The presence and persistence of mathematical difficulties for at least 6 months, despite the provision of interventions was also included among the criteria. Moreover, it was confirmed that the learning difficulties were not better accounted for by intellectual disabilities or by psychosocial adversity and inadequate educational instruction. The presence of physical, sensory, or neurological disorders was excluded. All students were Caucasian and spoke Italian fluently. Their intellectual abilities, except for those associated with mathematics, were within the average range according to the WISC-IV (Wechsler, 2003). According to their teachers, each of our participants had grown up in an adequate socio-cultural environment.

**Table 1:** Distribution of the children in the two conditions (experimental and control) by school year, gender, and age.

School year	Experimental group					Control group				
	Age (months)					Age (months)				
*N*	Male	Female	*M*	*SD*	*N*	Male	Female	*M*	*SD*
Primary school third	3	0	3	100.53	2.15	2	0	2	109.82	9.83
Primary school fourth	5	0	5	107.10	1.82	5	0	5	110.39	5.55
Primary school fifth	9	6	3	121.75	3.53	7	5	2	125.30	7.56
Secondary school first	9	2	7	133.16	4.11	10	3	7	133.92	7.45
Secondary school second	4	3	1	146.79	3.12	3	2	1	149.76	0.69

The participants were classified by age, school year, and gender. They were randomly assigned to two conditions: 30 students in the experimental training condition received the specialized mathematics training at the time of the study; and 27 in the control condition followed a general educational support program while waiting to receive the specialized training (more details can be found in the “Control condition training” section). Table 1 summarizes the children’s distribution across the 5 school years and for each treatment condition. There were no significant differences between the experimental and the control groups in age, *t*(55) = −0.88, *p* = 0.38, IQ scores, *t*(55) = −0.12, *p* = 0.90, gender distribution, *χ*^2^(1) = 0.0008, *p* = 0.97, or initial mathematical performance (all values of *p* > 0.05 across tests). Demographical data of the full sample can be found in the Supplementary Table A.

We received the necessary approval from the parents and schools for all the students involved in this investigation.

### Procedure

The intervention took place at “Polo Apprendimento” centers (see “Participants” section for details), and it was provided individually in a quiet room by a psychologist specialized in learning disabilities. The psychologists were observed and supervised by one of the authors every 3 weeks.

The study involved the following phases:

1. *Pre-training assessment*: Individual learning profiles were evaluated in order to identify the areas of greatest deficiency on which to focus the training.
2. *Training*: 30 weekly 1-h sessions with the psychologist and 15 min every day at home for the entire duration of the training program.
3. *Post-training assessment* (efficacy analysis): Post-treatment assessments were performed 1 week after the end of the training program.
4. *Follow-up*: Only for the experimental group, we performed follow-up assessments 2 months after the end of the training program.

### Structure of the Experimental Training Program

Children assigned to the experimental condition completed tasks differentiated and adapted to their individual difficulties, based on the assessment of their learning profiles. Each student started from the areas in which he/she struggled the most with exercises that contained a substantial amount of scaffolding, which was gradually removed. The psychologist organized the activities in order to progress step by step, respecting the child’s specific deficits in the various mathematical areas and at the same time, taking into consideration the child’s competences. This method derives from concept of zone of proximal development of Vygotsky (1931), defined as the distance between the actual developmental level as determined by independent problem-solving and the level of potential development as determined through problem-solving under adult guidance, or in collaboration with more capable peers. Notably, the activities were not just an extension of the math curriculum taught at school, and they were adjusted to each individual’s needs.

The instructional sessions were organized as follows:

1. *Presentation of the task and explanations of the final goal*: Each task was presented through various modalities (phonological, visual, and analogical) in order to increase the chance of students choosing their prefer modality and guarantee a better access and coding of the task. The aim was to enhance the students’ understanding of the activities’ meaning.
2. *Working on the material*: The psychologist introduced various strategies that the students could adopt or modify in order to suit their needs (constantly referring to their independent management of their learning processes). The material included both digital and pencil-and-paper tools.
3. *Discussion*: The student was invited to compare the easiness and accessibility of the various strategies with the psychologist’s guidance in order to reinforce metacognitive abilities.
4. *Summarizing*: At the end of each session, the child and then the operator made an overall recapitulation of the activities and strategies learned.
5. *Self-assessment*: conducted by the students, considering metacognitive and motivational components.
6. *Practicing*: The strategies learned were practiced in a coordinated, continuous, and contextualized manner. The procedural aspects were integrated with reasoning and metacognitive processes with the aim of helping the students understand how to employ the newly learned activities to support their arithmetic competence.

### Contents of the Experimental Training Program

The experimental training primarily focused on supporting new strategies in fundamental calculation skills (mental and written calculations; Lucangeli et al., 2003, 2004) and basic concepts of number (numerical knowledge; Lucangeli et al., 2005), like in Re et al. (2014). Moreover, a Web App was specifically designed for promoting understanding and meaning, and then for automaticity in retrieving and using arithmetic facts (e.g., “5 × 6” or “3 × 4”; Poli et al., 2006). A great effort was made to avoid mere learning by heart without understanding (like a nursery rhyme). Arithmetic facts are seen as being fundamental to the acquisition of calculation skills. The final goal was then to reduce the cognitive load when performing more complex calculations.

### Characteristics of the Web App “I bambini contano”

**Figure 1:**
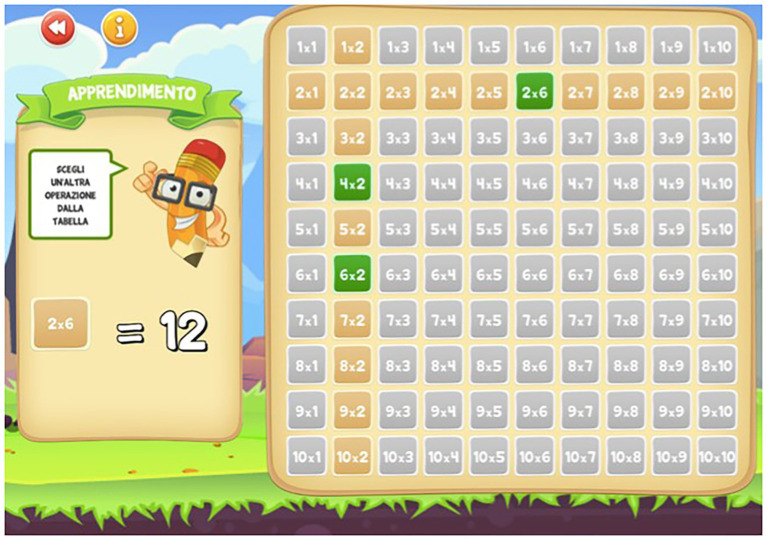
Example of learning phase of the Web App “I bambini contano.”

**Figure 2:**
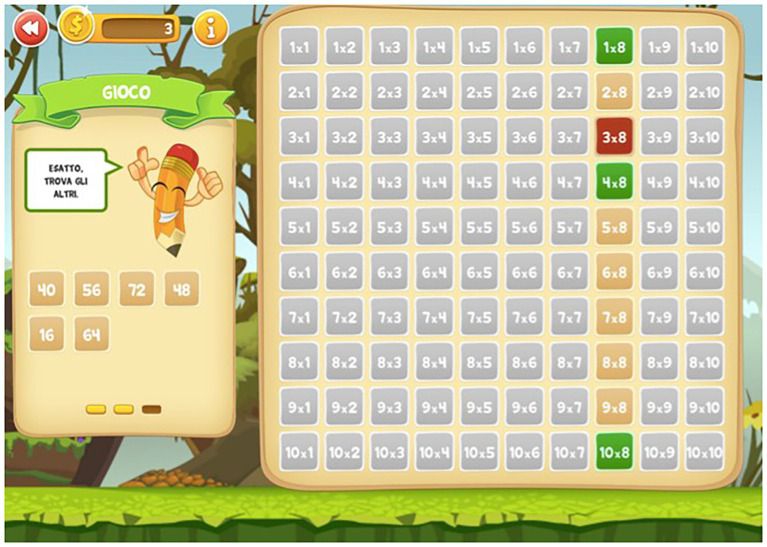
Example of game phase of the Web App “I bambini contano.”

**Figure 3:**
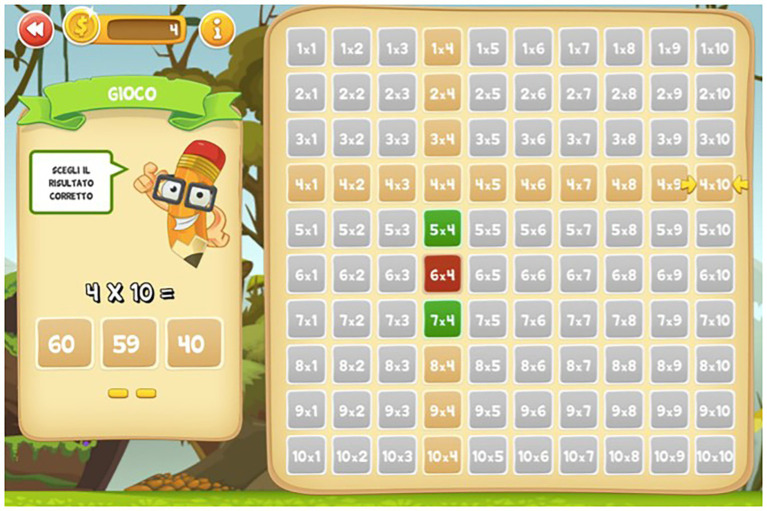
Example of game phase of the Web App “I bambini contano.”

The Web App consisted in a table of Pythagoras and included three levels of exercises for each of the 10 multiplication tables: in the first level, called “learning” (Figure 1), the child selects a given operation in the table, and the result appears on the screen (e.g., 2 × 6 = 12); and in the second level, called “exercise” (Figure 2), the child has to find the correct result of the operation (e.g., 2 × 4 = ?). In this level, there are no restrictions on the number of responses the child can attempt to provide; and in the third level, called “game” (Figure 3), the child has to find the correct result but only has three possibilities (e.g., 4 × 10 = ?).

For every multiplication table, there are always two conditions: the screen shows the operation (e.g., 2 × 4); the student has to find the result (e.g., 8) or the screen shows the result (e.g., 8); and the student has to find the corresponding operation (e.g., 2 × 4 or 4 × 8). Moreover, there are three possible modalities for every multiplication table: (a) the student has to write the result (or the corresponding operation); (b) the student has to find the result (or the corresponding operation) choosing from among three possibilities (e.g., 2 × 4 → 4, 8, and 18); (c) the student has to drag the correct result choosing from among several possibilities, and place the result in the correct position in the table of Pythagoras.

In total, the Web App offers 180 types of exercises.

### Control Training Program

Students assigned to the control group were supported by educators specialized in the training of children with learning disabilities. They worked over an equivalent amount of time, number of sessions, and on the same topics as the experimental group (i.e., concepts of number, automaticity in retrieving and using arithmetic facts, and mental and written calculation tasks). In this condition, however, the activities represented an extension of the math curriculum taught at school; they were differentiated by grade level and not tailored to the mathematical learning profile of each child. Moreover, the children did not use the Web App. To ensure that the educators closely follow the training topics and actually served a supporting role, these activities were monitored and supervised by one of the authors of the present paper.

### Fidelity of Implementation

During the experimental and control training programs, the operator kept a detailed written daily record of the activities carried out in each session. Furthermore, a written record was preserved for each of the supervision sessions held in the control condition. The record considered both the contents and the structure of each session. Correspondence between the observed activities and intended components of the lessons was approximately 90%.

### Dependent Measures

The assessments were carried out using the calculation abilities- metacognitive team (AC-MT) that is one of the most frequently used batteries for assessing calculation abilities in children from primary (AC-MT 6–11; Cornoldi et al., 2002) and secondary schools (AC-MT 11–14; Cornoldi and Cazzola, 2004) in Italy. Test-retest reliability of the AC-MT 6–11 is *r* = 0.65 (mean for all subtests); inter-rater reliability ranges from *r* = 0.38 to *r* = 0.85 (Cornoldi et al., 2002). Test-retest reliability of the AC-MT 11–14 is *r* = 0.61 (mean for all subtests; Cornoldi and Cazzola, 2004). The structure of the battery is the same for primary and secondary schools, but the difficulty of exercises changes according to grade level.

Pre‐ to post-training improvement as well as the students’ performance in the follow-up were specifically measured in terms of arithmetic fact skills, which were the main target of the training program. Additionally, we measured for improvement on mental calculation and written operation tasks, hypothesizing a possible transfer to these areas. A detailed description of the AC-MT tasks, from which the dependent measures were derived, is presented below.

1. *Arithmetic facts*: This task is used to investigate students’ automatic access to combinations of numbers without interim purposive calculation procedures. Examples of arithmetic facts are simple operations, such as 6 × 3 = 18; 8 + 2 = 10; and 10 − 5 = 5. The items include addition, subtraction, and multiplication, presented verbally and allowing 5 s to answer each item. There were 12 operations for primary school participants and 24 operations for secondary school students. Responses are scored for *percentage of accuracy* (i.e., percentage of correct answers). The difficulty of the operations varies according to the school level (e.g., primary school: 6 × 6, 28 − 8; secondary school: 7 × 8; 83 + 7).
2. *Mental calculation*: Students are asked to perform mental calculations. Two dependent variables are considered: *response time* and *percentage of accuracy* (i.e., percentage of correct answers). The response time is measured from the moment the examiner finishes uttering the numbers in the operation to the moment when the child begins answering. The time limit for each calculation is 30 s. There were six operations for primary school participants and four operations for secondary school students. The difficulty of the operations varies according to the school level (e.g., primary school: 19 − 6, 12 + 8; secondary school: 48:12, 14 × 2).
3. *Written calculation*: This task assesses the student’s ability to apply the procedures needed to complete written operations (e.g., addition, subtraction, and multiplication). Responses are scored as *percentage of accuracy* (i.e., percentage of correct answers). The test included eight operations for both primary and secondary school students. The difficulty of the operations varies according to the school level (e.g., primary school: 14 + 7; 15 − 8; secondary school: 4,724, 6 + 863, 9; 13,596 − 9,098).

### Data Analysis

The data were analyzed using a repeated measures analysis of variance (ANOVA) with a group (experimental vs. control) as between subject’s factor and time (pre-training vs. post-training) as within subject’s factor. Moreover, for the experimental group only, a one-way ANOVA was used to compare the students’ performance at three different time points (pre-training, post-training, and follow-up). In addition, we analyzed the results with a qualitative approach following the guidelines issued by the Consensus Conference (2010) on Learning Disabilities, which define a positive change of at least one standard deviation to represent improvement; therefore, we considered the percentage of participants in each group who met this criterion. Given the sample size and in order to increment the statistical power, we decided to include within each condition/group children from both primary and secondary school levels. Separate analysis for each school level can be also found in the Supplementary Materials.

## Results

### Efficacy of the Treatment

Table 2 displays the descriptive statistics for both the experimental and control groups. The ANOVA with arithmetic facts as dependent measure yielded a significant main effect of time, *F*(1,55) = 22.32, *p* < 0.0001, *η*^2^*_p_* = 0.28, and a significant interaction between factors, *F*(1,55) = 13.29, *p* < 0.001, *η*^2^*_p_* = 0.19. The same pattern was observed for written calculation, and there was a significant main effect of time, *F*(1,55) = 8.53, *p* < 0.005, *η*^2^*_p_* = 0.13, and a modest interaction between group and time factors, *F*(1,55) = 3.94, *p* = 0.05, *η*^2^*_p_* = 0.07. In both cases, the main effect of time reflected an improvement from pre‐ to post-training assessments, and the interaction indicated a differential pre-post gain in favor of the experimental condition. For mental calculation accuracy and mental calculation time, we observed no significant main effects and no interactions between factors. Separate analyses for students in primary and secondary schools revealed similar effects for arithmetic facts in both groups and effects in mental and written calculations for the primary school only (see Supplementary Tables C and D).

**Table 2:** Descriptive data of experimental group and control group at pre-training, post-training, and follow-up.

Variable		Experimental group		Control group		ANOVA time (Pre-post) * Group (Exp-Ctrl)	
		(*N* = 30)		(*N* = 27)			
		Age range (8.2–12.6); Mean IQ = 97		Age range (8.5–12.5); Mean IQ = 97			
		*M*	*SD*	*M*	*SD*		
Mental calculation	Pre	55.0	29.3	54.6	34.8	Time	*F*(1,55) = 1.93, *η*^2^*_p_* = 0.03, *p* > 0.05
	Post	68.3	26.7	53.4	37.4	Group	*F*(1,55) = 1.09, *η*^2^*_p_* = 0.019, *p* > 0.05
	Follow-up	69.2	29.5	-	-	Time * Group	*F*(1,55) = 2.79, *η*^2^*_p_* = 0.05, *p* > 0.05
Mental calculation time	Pre	93.6	48.4	109.3	51.2	Time	*F*(1,55) = 0.54, *η*^2^*_p_* = 0.01, *p* > 0.05
	Post	98.0	62.9	118.7	78.0	Group	*F*(1,55) = 1.89, *η*^2^*_p_* = 0.03, *p* > 0.05
	Follow-up	88.4	47.3	-	-	Time * Group	*F*(1,55) = 0.07, *η*^2^*_p_* = 0.001, *p* > 0.05
Arithmetical facts	Pre	33.7	19.0	36.4	19.3	Time	***F*(1,55) = 22.32, *η***^**2**^_***p***_ **= 0.28, *p* < 0.0001**
	Post	57.9	19.2	39.5	24.1	Group	*F*(1,55) = 2.94, *η*^2^*_p_* = 0.05, *p* > 0.05
	Follow-up	55.8	23.7	-	-	Time * Group	***F*(1,55) = 13.29, *η***^**2**^_***p***_ **= 0.19, *p* < 0.001**
Written calculation	Pre	45.0	24.5	39.8	22.2	Time	***F*(1,55) = 8.53, *η***^**2**^_***p***_ **= 0.13, *p* < 0.005**
	Post	59.6	29.1	42.6	24.1	Group	*F*(1,55) = 3.43, *η*^2^*_p_* = 0.06, *p* > 0.05
	Follow-up	56.3	31.3	-	-	Time * Group	***F*(1,55) = 3.94, *η***^**2**^_***p***_ **= 0.07, *p* = 0.05**

The bold values are significative results.

### Follow-Up Analysis

Table 2 displays the descriptive statistics of the follow-up evaluation of the experimental group. For arithmetic facts, the ANOVA over the three measures (pre-training, post-training, and follow-up) showed a main effect of time, *F*(2,58) = 20.71, *p* < 0.0001, *η*^2^*_p_* = 0.42, due to significant differences between the pre‐ and post-training assessments (*p* < 0.0001), and also between the pre-training and follow-up measures (*p* < 0.0001). There were no additional changes between the post-training and follow-up measures (*p* = 0.86).

The same analysis carried out for written calculation showed a significant main effect of time, *F*(2,58) = 4.29, *p* = 0.01, *η*^2^*_p_* = 0.13, due to significant changes between the pre‐ and post-training measures (*p* = 0.01). There were no significant differences between the pre-training and follow-up measures (*p* = 0.15) or between the post-training and follow-up assessments (*p* = 0.77). There were no significant effects for mental calculation accuracy or mental calculation time.

### Qualitative Change

To further evaluate the validity of the training program, we implemented previously used criteria (Re et al., 2014) based on the guidelines issued by the Consensus Conference (2010) on Learning Disabilities. A significant qualitative change was defined as a progression of at least one standard deviation. Transition from more problematic to less problematic levels suggests a significant improvement. This analysis was applied because a change that might not be statistically significant at the group level could sometimes be of value in qualitative terms at the individual level; this type of analysis reveals improvements that could go unnoticed when group averages are analyzed, but may be very important for the individual student.

Table 3 displays the effect sizes (Cohen’s *d*) of the comparisons between the percentages of participants meeting the qualitative criteria for a positive change in each of the groups (experimental vs. control). Based on these criteria, the experimental training program improved students’ performance compared with that of controls in all parameters.

**Table 3:** Qualitative comparison: number and frequencies of children who changed at least one SD from the pre to the post-training, in the experimental training and control training conditions.

Variable	Training		Cohen’s *d*
	Experimental	Control	
Mental calculation	17 (56.67%)	5 (18.52%)	1.1
Mental calculation time	10 (33.33%)	5 (18.52%)	0.4
Arithmetical facts	20 (66.67%)	2 (7.41%)	4.4
Written calculation	13 (43.33%)	3 (11.11%)	0.9

## Discussion

The present study evaluated the effectiveness of a shortened specialized and digitally supported training program for enhancing numerical skills in primary and secondary school students with MD. We randomly assigned children to the training and control groups and controlled for a variety of confounding factors. At pre-test, we made sure that the age, gender, general intelligence (IQ), and mathematical performance of the experimental group were comparable to those of the control group. The experimental group followed a mathematical training program tailored to each individual’s mathematical learning profile and used a Web App tool specifically designed for practicing arithmetic facts, i.e., one of the participant’s severely impaired numerical skills (ranging from 33 to 36% of accuracy; see Table 2) at home. The control sample carried out activities designed to help them complete and understand mathematical tasks as a part of their school curriculum, but they did not use the Web App. The control and experimental training programs involved the same contents, in the same settings, for the same amount of time and number of training sessions. Moreover, both activities were conducted under the supervision of the same experimenter.

In both groups, the pre-post group evaluations showed an improvement of the students’ performance in arithmetic facts and written calculation tasks. Noticeably, the experimental group demonstrated greater improvements than the control group, suggesting an advantage for the specialized program that incorporated the use of the Web App.

This improvement is not surprising, given that the basic structure of the Web App used by the experimental group, but not by the control group, is rooted in the learning and practicing of multiplication tables, which constituted a primary intervention goal of the program. Developing the ability to access arithmetic facts quickly and automatically is crucial, given that this mechanism is frequently compromised in children with dyscalculia (Garnett and Fleischner, 1983; Goldman, 1989; Koscinski and Gast, 1993; Lucangeli and Cornoldi, 2007), it is an essential building block in the construction of more advanced mathematical skills (Pressley, 1986; Koscinski and Gast, 1993), and it contributes to reducing mathematics anxiety (Wittman et al., 1998).

The results of our study are thus consistent with the current body of research in two important ways. First is by reporting the advantages of digital tools with respect to traditional practices (e.g., pencil-and-paper activities, flashcards, etc.) for the automation of arithmetic facts in children with MD (Koscinski and Gast, 1993; Burns et al., 2012; Kiger et al., 2012; Aragón‐Mendizábal et al., 2017; Mera et al., 2019; Benavides-Varela et al., 2020a). And second, by replicating the results obtained in a previous study conducted by the same authors (Re et al., 2014), is underlining the importance of developing training programs tailored on the basis of the child’s mathematical learning profile rather than adopting a specific mathematics teaching procedure, which has been the case in the majority of the published studies (e.g., Gersten et al., 2009; Dennis et al., 2016).

The present research also extends previous studies by indicating that the introduction of a digital tool at home, aiming at automating arithmetic facts within a customized intervention framework enables a significant reduction in the number of face-to-face training sessions. Moreover, the results show that this specialized training program can continue to be effective for up to 2 months after it has ended. In our previous study (Re et al., 2014), participants attended 48 one-hour training sessions, whereas in the current one, 30 sessions were sufficient to obtain effective and sustained results. Besides involving individualized practice (Nelson et al., 2013), game-based interventions have the general advantage of making children more motivated and increasing the opportunities for individual practice at home. Learners benefit from shorter training times, as they can catch up to achieve their educational goals more quickly, with redundant benefits arising from their increased motivation and self-esteem. The fact that the effects of the training are preserved over time also suggests that the numerical facts are consolidated thanks to the use of the Web App at home.

As in the previous study, we found that, at the group level, the mathematical area most resistant to improvement is mental calculation. This area is probably the most difficult for children with MD and especially for those with severe difficulties. Indeed, in the previous study, we only reported an improvement in mental calculation accuracy among children with mild mathematical difficulties, while those with severe mathematical difficulties did not improve. This result appears to support the idea that children with severe mathematical difficulties or with dyscalculia are resistant to training in some mathematical areas, such as mental calculation (Landerl et al., 2004; Lucangeli and Mammarella, 2010; Re et al., 2014).

Still, we were able to detect an improvement in scores on all numerical tasks, including mental calculation, using a validated qualitative criterion that takes into account progress at the individual level. From this type of analysis, it emerged more clearly that more children in the experimental group improved as compared to the corresponding control group. This means that significantly fewer students remained at risk for math failure after taking part in the experimental intervention.

### Limitations

A first limitation of this study is that we included children from both primary (6–10 years old) and secondary (11–13 years old) schools. This was a limitation, because the size of the primary and secondary school groups was neither comparable nor sufficient to carry out separate analyses. Separate analysis for primary and secondary school children (see Supplementary Materials), showed significant improvements in all the tasks (mental, written calculation, and arithmetic facts) for primary school children and in arithmetic fact only for secondary school children. However, the reduced sample size and the low effect sizes in secondary school prevent us from drawing definitive conclusions. Future studies extending to more participants in secondary school should be able to shed light on this fundamental issue.

Another limitation that might have influenced the outcome of this study was the professional bias of the specialists working with the children. Indeed, psychologists worked with children in the experimental group and educators were in charge of the intervention for the control group. In order to reduce this possible bias, our center selected educators with a great deal of experience in working with children with mathematical difficulties. All the educators involved had completed a University specialization course in learning disabilities, psychopathology and/or worked with children with learning disabilities for many years. In addition, the educators and the psychologists involved in the study were all constantly supervised by the psychologist author of the present paper.

Whether the digital tool would be as effective if implemented without the explicit guidance of a psychologist or specialist is open to question. Since the Web App combines game-based and educational features, it could be directly proposed by parents (see Benavides-Varela et al., 2016; for other examples of family activities that can boost early numerical knowledge) or by teachers in formal educational settings both for individual and group activities and for children with or without LD.

Finally, because of ethical concerns, we could not add a passive control group in the current study (children with MD in an eventual passive control group must have waited for several months without receiving any support, which could have increased their anxiety and placed them under severe risk of school failure). Thus, it is possible that some of the effects observed (i.e., time effects) could have been, at least in part, due to maturation rather than training. In order to minimize this risk, we used age appropriate tests in each of the assessments. In fact, AC-MT includes tasks with various levels of difficulty determined by the school level and also the period of the academic year in which the assessment is performed (beginning, middle-term, or end of the academic year), thus allowing for a fine graded appropriate assessment.

## Conclusion

To summarize, our findings enable us to draw two conclusions: first, a specific training program adapted according to each child’s cognitive profile is a better solution for effective training purposes, and the results obtained are generally durable; and second, the use of the Web App “I bambini contano” (“Children count”) at home, which was specifically designed for training arithmetic facts can produce positive changes in children of primary and secondary school age and can significantly reduce training time, without reducing the effectiveness of the intervention.

## Data Availability Statement

The raw data supporting the conclusions of this article will be made available by the authors, without undue reservation.

## Ethics Statement

The studies involving human participants were reviewed and approved by Institutional Ethics Committee approval of the School of Psychology at Padua University. Written informed consent to participate in this study was provided by the participants’ legal guardian/next of kin.

## Author Contributions

AR developed the project, designed the Web-App, and wrote the manuscript. SB-V analyzed the data and wrote the manuscript. MP performed the training and coordinate the training part of the project. MD performed the training. DL supervised the project. All authors contributed to the article and approved the submitted version.

### Conflict of Interest

The authors declare that the research was conducted in the absence of any commercial or financial relationships that could be construed as a potential conflict of interest. The handling editor declared a shared affiliation with one of the authors AR.
