# Supplementary material for: Response to a Specific and Digitally Supported Training at Home for Students With Mathematical Difficulties
Source: Front Psychol. 2020 Aug 31;11:2039. doi: 10.3389/fpsyg.2020.02039 (PMC7489095; doi:10.3389/fpsyg.2020.02039)
Supplement: Supplementary file 1 [file Data_Sheet_1.docx]

**SUPPLEMENTARY MATERIALS**

**TABLE A – DEMOGRAPHIC DATA**

| **Demographic Variables** | | | | | |
| --- | --- | --- | --- | --- | --- |
| **ID** | **Group** | **School Level** | **Gender** | **Age** | **IQ** |
| 1 | Experimental | Primary | F | 8,3 | 80 |
| 2 | Experimental | Primary | F | 8,3 | 74 |
| 3 | Experimental | Primary | F | 8,6 | 92 |
| 4 | Experimental | Primary | F | 9,2 | 104 |
| 5 | Experimental | Primary | F | 8,8 | 97 |
| 6 | Experimental | Primary | F | 8,8 | 99 |
| 7 | Experimental | Primary | F | 8,9 | 103 |
| 8 | Experimental | Primary | F | 8,9 | 97 |
| 9 | Experimental | Primary | M | 10,0 | 112 |
| 10 | Experimental | Primary | M | 10,1 | 109 |
| 11 | Experimental | Primary | M | 10,4 | 110 |
| 12 | Experimental | Primary | M | 10,1 | 93 |
| 13 | Experimental | Primary | F | 10,8 | 95 |
| 14 | Experimental | Primary | F | 10,1 | 107 |
| 15 | Experimental | Primary | M | 9,8 | 93 |
| 16 | Experimental | Primary | F | 9,9 | 103 |
| 17 | Experimental | Primary | M | 10,2 | 88 |
| 18 | Experimental | Secondary | M | 11,6 | 97 |
| 19 | Experimental | Secondary | F | 11,0 | 94 |
| 20 | Experimental | Secondary | M | 10,8 | 84 |
| 21 | Experimental | Secondary | F | 10,8 | 80 |
| 22 | Experimental | Secondary | F | 11,8 | 102 |
| 23 | Experimental | Secondary | F | 11,0 | 112 |
| 24 | Experimental | Secondary | F | 10,9 | 87 |
| 25 | Experimental | Secondary | F | 11,1 | 113 |
| 26 | Experimental | Secondary | F | 10,9 | 120 |
| 27 | Experimental | Secondary | M | 12,6 | 83 |
| 28 | Experimental | Secondary | F | 12,0 | 88 |
| 29 | Experimental | Secondary | M | 12,2 | 93 |
| 30 | Experimental | Secondary | M | 12,1 | 92 |
| 1 | Control | Primary | F | 9,7 | 115 |
| 2 | Control | Primary | F | 8,6 | 101 |
| 3 | Control | Primary | F | 8,5 | 120 |
| 4 | Control | Primary | F | 9,1 | 116 |
| 5 | Control | Primary | F | 9,4 | 96 |
| 6 | Control | Primary | F | 9,7 | 91 |
| 7 | Control | Primary | F | 9,4 | 102 |
| 8 | Control | Primary | M | 9,7 | 92 |
| 9 | Control | Primary | M | 10,4 | 109 |
| 10 | Control | Primary | M | 10,7 | 86 |
| 11 | Control | Primary | M | 10,3 | 92 |
| 12 | Control | Primary | F | 9,9 | 102 |
| 13 | Control | Primary | F | 10,4 | 86 |
| 14 | Control | Primary | M | 11,6 | 96 |
| 15 | Control | Secondary | M | 10,3 | 92 |
| 16 | Control | Secondary | M | 10,5 | 99 |
| 17 | Control | Secondary | F | 12,3 | 75 |
| 18 | Control | Secondary | M | 11,3 | 95 |
| 19 | Control | Secondary | F | 11,4 | 99 |
| 20 | Control | Secondary | F | 12,1 | 97 |
| 21 | Control | Secondary | F | 11,1 | 86 |
| 22 | Control | Secondary | F | 10,9 | 71 |
| 23 | Control | Secondary | F | 11,0 | 95 |
| 24 | Control | Secondary | F | 10,9 | 101 |
| 25 | Control | Secondary | M | 12,5 | 119 |
| 26 | Control | Secondary | F | 12,5 | 96 |
| 27 | Control | Secondary | M | 12,4 | 92 |

**TABLE B - PRE-TRAINING DATA**

|  | **Scorings at Pre-test** | | | |
| --- | --- | --- | --- | --- |
| **ID** | **Mental Calculation-Accuracy** | **Mental-Calculation-Time** | **Arithmetic Facts** | **Written-Calculation** |
| 1 | 66,7 | 86,0 | 16,7 | 50,0 |
| 2 | 66,7 | 78,0 | 33,3 | 50,0 |
| 3 | 33,3 | 84,0 | 33,3 | 75,0 |
| 4 | 100,0 | 35,0 | 58,3 | 50,0 |
| 5 | 100,0 | 54,0 | 33,3 | 62,5 |
| 6 | 50,0 | 95,0 | 33,3 | 50,0 |
| 7 | 66,7 | 71,0 | 41,7 | 75,0 |
| 8 | 83,3 | 79,0 | 16,7 | 50,0 |
| 9 | 66,7 | 50,0 | 25,0 | 87,5 |
| 10 | 50,0 | 53,0 | 25,0 | 50,0 |
| 11 | 33,3 | 122,0 | 75,0 | 87,5 |
| 12 | 83,3 | 122,0 | 8,3 | 50,0 |
| 13 | 66,7 | 99,0 | 25,0 | 50,0 |
| 14 | 33,3 | 135,0 | 33,3 | 62,5 |
| 15 | 33,3 | 139,0 | 8,3 | 25,0 |
| 16 | 66,7 | 98,0 | 41,7 | 25,0 |
| 17 | 0,0 | 90,0 | 8,3 | 25,0 |
| 18 | 0,0 | 170,0 | 16,7 | 0,0 |
| 19 | 16,7 | 211,0 | 16,7 | 50,0 |
| 20 | 50,0 | 125,0 | 33,3 | 37,5 |
| 21 | 16,7 | 163,0 | 16,7 | 12,5 |
| 22 | 83,3 | 53,0 | 58,3 | 37,5 |
| 23 | 83,3 | 168,0 | 33,3 | 62,5 |
| 24 | 25,0 | 62,0 | 45,8 | 87,5 |
| 25 | 25,0 | 144,0 | 12,5 | 0,0 |
| 26 | 100,0 | 22,0 | 62,5 | 50,0 |
| 27 | 75,0 | 16,0 | 75,0 | 12,5 |
| 28 | 25,0 | 75,0 | 45,8 | 25,0 |
| 29 | 75,0 | 22,0 | 25,0 | 12,5 |
| 30 | 75,0 | 88,0 | 54,2 | 37,5 |
| 1 | 66,7 | 70,0 | 50,0 | 25,0 |
| 2 | 33,3 | 150,0 | 33,3 | 25,0 |
| 3 | 83,3 | 115,0 | 58,3 | 87,5 |
| 4 | 100,0 | 70,0 | 25,0 | 50,0 |
| 5 | 100,0 | 80,0 | 8,3 | 50,0 |
| 6 | 50,0 | 170,0 | 25,0 | 50,0 |
| 7 | 50,0 | 17,0 | 66,7 | 75,0 |
| 8 | 100,0 | 56,0 | 83,3 | 50,0 |
| 9 | 100,0 | 70,0 | 41,7 | 50,0 |
| 10 | 100,0 | 40,0 | 41,7 | 50,0 |
| 11 | 50,0 | 102,0 | 25,0 | 37,5 |
| 12 | 66,7 | 72,0 | 50,0 | 25,0 |
| 13 | 0,0 | 220,0 | 16,7 | 75,0 |
| 14 | 50,0 | 76,0 | 33,3 | 50,0 |
| 15 | 50,0 | 100,0 | 25,0 | 37,5 |
| 16 | 16,7 | 211,0 | 16,7 | 50,0 |
| 17 | 16,7 | 163,0 | 16,7 | 12,5 |
| 18 | 83,3 | 53,0 | 58,3 | 37,5 |
| 19 | 83,3 | 168,0 | 33,3 | 62,5 |
| 20 | 0,0 | 118,0 | 16,7 | 0,0 |
| 21 | 50,0 | 125,0 | 58,3 | 37,5 |
| 22 | 50,0 | 185,0 | 12,5 | 12,5 |
| 23 | 0,0 | 100,0 | 8,3 | 12,5 |
| 24 | 100,0 | 120,0 | 50,0 | 62,5 |
| 25 | 50,0 | 100,0 | 50,0 | 25,0 |
| 26 | 0,0 | 101,0 | 41,7 | 12,5 |
| 27 | 25,0 | 99,0 | 37,5 | 12,5 |

**TABLE C – TRAINING EFFECTS FOR PRIMARY SCHOOL ONLY**

|  |  |  |  |  |  |  |  |
| --- | --- | --- | --- | --- | --- | --- | --- |
|  |  | Experimental Group | | Control Group | | **ANOVA Time (Pre-post) * Group(Exp-Ctrl)** | |
|  |  | (N=17) | | (N=14) | |  |  |
|  |  | Age range (8.2-10.7) | | Age range (8.5-11.6) | |  |  |
| *Variable* |  | M | SD | M | SD |  |  |
| Mental Calculation | Pre | 58,8 | 26,4 | 67,9 | 31,0 | Time | F(1,29)=.97, η2p =.03, p >.05 |
|  | Post | 73,5 | 23,6 | 61,9 | 34,2 | Group | F(1,29)=.02, η2p =.001, p >.05 |
|  | Follow-up | 76,5 | 19,6 | - | - | Time * Group | **F(1,29)=5.43, η2p =.16, p <.05** |
| Mental Calculation time | Pre | 87,6 | 30,1 | 93,4 | 54,3 | Time | F(1,29)=2.93, η2p =.09, p >.05 |
|  | Post | 99,1 | 66,0 | 121,2 | 94,6 | Group | F(1,29)=.48, η2p =.02, p >.05 |
|  | Follow-up | 83,7 | 37,0 | - | - | Time * Group | F(1,29)=.50, η2p =.02, p >.05 |
| Arithmetical facts | Pre | 30,4 | 17,7 | 39,9 | 20,5 | Time | **F(1,29)=19.62, η2p =.40, p <.0001** |
|  | Post | 59,3 | 19,5 | 39,3 | 21,3 | Group | F(1,29)=2.93, η2p =.09, p >.05 |
|  | Follow-up | 54,4 | 24,5 | - | - | Time * Group | **F(1,29)=21.30, η2p =.42, p <.0001** |
| Written calculation | Pre | 54,4 | 19,2 | 50,0 | 19,0 | Time | **F(1,29)=10.23, η2p =.26, p <.005** |
|  | Post | 73,5 | 25,0 | 54,5 | 22,3 | Group | F(1,29)=.69, η2p =.02, p >.05 |
|  | Follow-up | 62,5 | 31,9 | - | - | Time * Group | **F(1,29)=3.95, η2p =.12, p =.05** |

**TABLE D – TRAINING EFFECTS FOR SECONDARY SCHOOL ONLY**

|  |  | Experimental Group | | Control Group | | **ANOVA Time (Pre-post) * Group(Exp-Ctrl)** | |
| --- | --- | --- | --- | --- | --- | --- | --- |
|  |  | (N=13) | | (N=13) | |  |  |
|  |  | Age range (10.8-12.6) | | Age range (10.3-12.5) | |  |  |
| *Variable* |  | M | SD | M | SD |  |  |
| Mental Calculation | Pre | 50,0 | 33,2 | 40,4 | 34,0 | Time | F(1,24)=.89, η2p =.35, p >.05 |
|  | Post | 61,5 | 30,0 | 44,2 | 39,7 | Group | F(1,24)=1.56, η2p =.06, p >.05 |
|  | Follow-up | 59,6 | 37,6 | - | - | Time * Group | F(1,24)=.22, η2p =.009, p >.05 |
| Mental Calculation time | Pre | 101,4 | 65,8 | 126,4 | 43,4 | Time | F(1,24)=.25, η2p =.01, p >.05 |
|  | Post | 96,5 | 61,2 | 115,9 | 58,9 | Group | F(1,24)=1.72, η2p =.07, p >.05 |
|  | Follow-up | 94,5 | 59,2 | - | - | Time * Group | F(1,24)=.03, η2p =.001, p >.05 |
| Arithmetical facts | Pre | 38,1 | 20,4 | 32,7 | 17,9 | Time | **F(1,24)=6.2, η2p =.20, p =.02** |
|  | Post | 56,1 | 19,4 | 39,8 | 27,8 | Group | F(1,24)=2.51, η2p =.09, p >.05 |
|  | Follow-up | 57,7 | 23,4 | - | - | Time * Group | F(1,24)=1.17, η2p =.05, p >.05 |
| Written calculation | Pre | 32,7 | 25,8 | 28,8 | 20,7 | Time | F(1,24)=.98, η2p =.04, p >.05 |
|  | Post | 41,3 | 24,1 | 29,8 | 19,5 | Group | F(1,24)=1.06, η2p =.04, p >.05 |
|  | Follow-up | 48,1 | 29,7 | - | - | Time * Group | F(1,24)=.63, η2p =.03, p >.05 |
